# Intimate Partner Violence Perpetration and PrEP Use Among Sexual Minority Men: The Mediating Roles of Internalized Homonegativity and PrEP Stigma

**DOI:** 10.1007/s10461-025-04802-y

**Published:** 2025-06-24

**Authors:** Erik D. Storholm, Jessica Randazzo, Chenglin Hong, Daniel E. Siconolfi, Carrie L. Nacht, Sarita D. Lee, Glenn J. Wagner

**Affiliations:** 1 School of Public Health, San Diego State University, 5500 Campanile Drive, Hepner Hall 114E, San Diego, CA 92182-4162 USA; 2 RAND Behavioral and Policy Sciences, Santa Monica, CA USA; 3 Department of Family Medicine, Center for HIV Identification, Prevention and Treatment Services, University of California Los Angeles, Los Angeles, CA USA; 4 School of Social Work, University of Connecticut, Hartford, CT USA; 5 Herbert Wertheim School of Public Health and Human Longevity Science, University of California, San Diego, La Jolla, CA USA

**Keywords:** Intimate partner violence (IPV), Sexual minority men (SMM), HIV pre-exposure prophylaxes (PrEP), Internalized homonegativity, PrEP stigma

## Abstract

Cisgender sexual minority men (SMM) report intimate partner violence (IPV) at rates comparable to or higher than heterosexual cisgender women, often linked to increased HIV risk. This study explores the relationship between IPV perpetration and pre-exposure prophylaxis (PrEP), considering the potential mediating effects of sexual orientation related minority stress and PrEP stigma. Utilizing baseline data from the Empowering Relationships and Opportunities for Safety (EROS) cohort, this cross-sectional study examined survey data from partnered cisgender SMM in the U.S. Through online and community recruitment, participants were assessed for IPV perpetration using a validated measure, while internalized homonegativity and related stigma were evaluated through standardized scales. Current PrEP use was assessed through self-report and confirmed through dried blood spot assays. Of the 500 participants, 125 (25%) reported IPV perpetration, with perpetration of identity-related IPV (e.g., threatening to out a partner to family or coworkers; telling partner to act straight) significantly higher among non-PrEP users; mean number of identity-related IPV perpetration items endorsed was 0.21 (SD = 0.53) among non-PrEP users compared to 0.02 (SD = 0.14) among PrEP users. Perpetration of identity-related IPV was positively correlated with internalized homonegativity (*r* =.19, *p* <.001), experienced sexual orientation discrimination (*r* =.15, *p* <.01), and PrEP stigma (*r* =.14, *p* <.001). A logistic regression mediation analysis found that, separately, internalized homonegativity (OR = 0.97, 95% CI [0.94–0.99]) and PrEP stigma (OR *=* 0.48, 95% CI [0.40, 0.57]) had significant associations with PrEP use, and that each mediated the relationship between perpetration of identity-related IPV and PrEP use as demonstrated by their significant indirect effects (OR *=* 0.88, 95% CI [0.78, 0.98] and (OR *=* 0.77, 95% CI [0.60, 0.98], respectively). The study underscores internalized homonegativity and PrEP stigma as critical mediators of the relationship between perpetration of identity-related IPV and PrEP use among SMM. Findings call for interventions aimed at reducing internalized homonegativity, PrEP stigma and enhancing PrEP access while addressing IPV dynamics. Future research should further delineate these pathways to inform culturally sensitive interventions promoting health equity among SMM.

## Introduction

Cisgender sexual minority men (SMM) experience intimate partner violence (IPV) at rates equal to or higher than those of heterosexual cisgender women [1, 2]. This violence can manifest in various forms, including physical, sexual, emotional, financial abuse, and social isolation [3]. IPV among SMM is associated with increased sexual risk behaviors, substance use, and susceptibility to HIV and other sexually transmitted infections (STIs) [4, 5]. Despite these associations, limited research has explored the pathways by which IPV affects engagement in HIV preventive behaviors among SMM, such as the use of pre-exposure prophylaxis (PrEP) [6, 7]. Indeed, a recent review of the limited literature suggests a complex relationship between IPV and PrEP utilization, underscoring the need for more research to better understand these dynamics and underlying mechanism to inform the development of future services and interventions [6]. 

SMM often experience stigma based on their sexual orientation and their sexual behavior [8, 9] and these experiences can become internalized [10, 11]. Internalized homonegativity, defined as the internalization of negative societal attitudes towards one’s own non-heterosexual orientation [12], may contribute to elevated rates of IPV perpetration among some SMM [13, 14]. For example, internalized homonegativity can result in pervasive feelings of shame and guilt, which may manifest as aggression directed toward same-sex partners who represent and witness the very attractions and behaviors that evoke such shameful emotions [14, 15]. Additionally, socialized gender role dynamics within SMM relationships may cause power imbalances, potentially exacerbating IPV [16–18]. Experiences of homophobia, discrimination, and violence can further compound these issues by impairing emotional regulation and healthy conflict management, thereby increasing vulnerability to IPV perpetration [19, 20]. 

Internalized homonegativity may also contribute to stigmatizing attitudes about the use of PrEP, or stereotypes about people who use PrEP as being promiscuous or taking sexual risks known as PrEP stigma [21], and has been shown to be associated with lower rates of PrEP use among SMM [7, 22–24]. When individuals harbor negative attitudes towards their own same-sex sexual attractions and behaviors, they may feel ashamed of engaging in health-promoting behaviors such as PrEP uptake that have been culturally associated with same-sex sexual behavior and have historically been marketed largely to SMM [21, 25–27]. This stigma may deter them from seeking information about PrEP, discussing PrEP with other SMM or their providers, and from attending organizations known to provide PrEP to SMM out of fear of judgment or association with negative stereotypes about SMM [21, 27, 28]. Consequently, internalized homonegativity not only has the potential to affect interpersonal dynamics, such as through perpetration of IPV, but may also undermine engagement with critical health interventions like PrEP, exacerbating HIV transmission risk within this population.

Minority Stress Theory posits that chronic stressors faced by marginalized groups, such as SMM, contribute to poorer health outcomes [29, 30]. This theory proposes that prejudice, discrimination, and stigma, in the social environment, creates a unique set of stressors that are not experienced by majority groups [25]. These stressors can lead to the internalization of homonegativity and increased mental health issues, substance use and vulnerability to IPV perpetration, as well as reduced engagement in HIV prevention strategies like PrEP [31, 32]. The psychological mediation framework of minority stress suggests that the link between IPV perpetration and PrEP use may be mediated by internalized homonegativity and other discriminatory experiences [13, 33, 34]. 

Despite these known challenges, little is known about how specific forms of IPV perpetration may relate to both internalized homonegativity and PrEP use among SMM. Few studies have addressed this intersection [6], highlighting an urgent need for further study in order to develop targeted, culturally sensitive interventions. Further, the disproportionately low rates of PrEP use and higher rates of HIV among SMM of color [35, 36] underscore the critical need for research in this area, particularly given the prevalence of IPV among SMM [1, 2]. Understanding and addressing the social and structural determinants of IPV and associated HIV risks must be prioritized to reduce these burdens and promote the overall health and well-being of SMM affected by IPV. The current analysis sought to fill this gap in research by assessing the relationship between different forms of IPV perpetration and PrEP use, and the potentially mediating role of internalized homonegativity, PrEP stigma, and other discriminatory experiences.

## Methods

### Study Design

This study employed a cross-sectional analysis using baseline data from the ongoing *Empowering Relationships and Opportunities for Safety* (EROS) longitudinal observational cohort study, designed to explore the role of IPV and HIV health behaviors among cisgender SMM who report being in relationships with other cisgender SMM [37]. Participants were initially recruited from a combination of online and SMM community settings and are being followed over a 24-month period, though only baseline data is considered here as the study is not scheduled to complete data collection until 2026. The study received approval from the Institutional Review Board at San Diego State University ensuring ethical compliance throughout.

### Participants

**Fig. 1:**
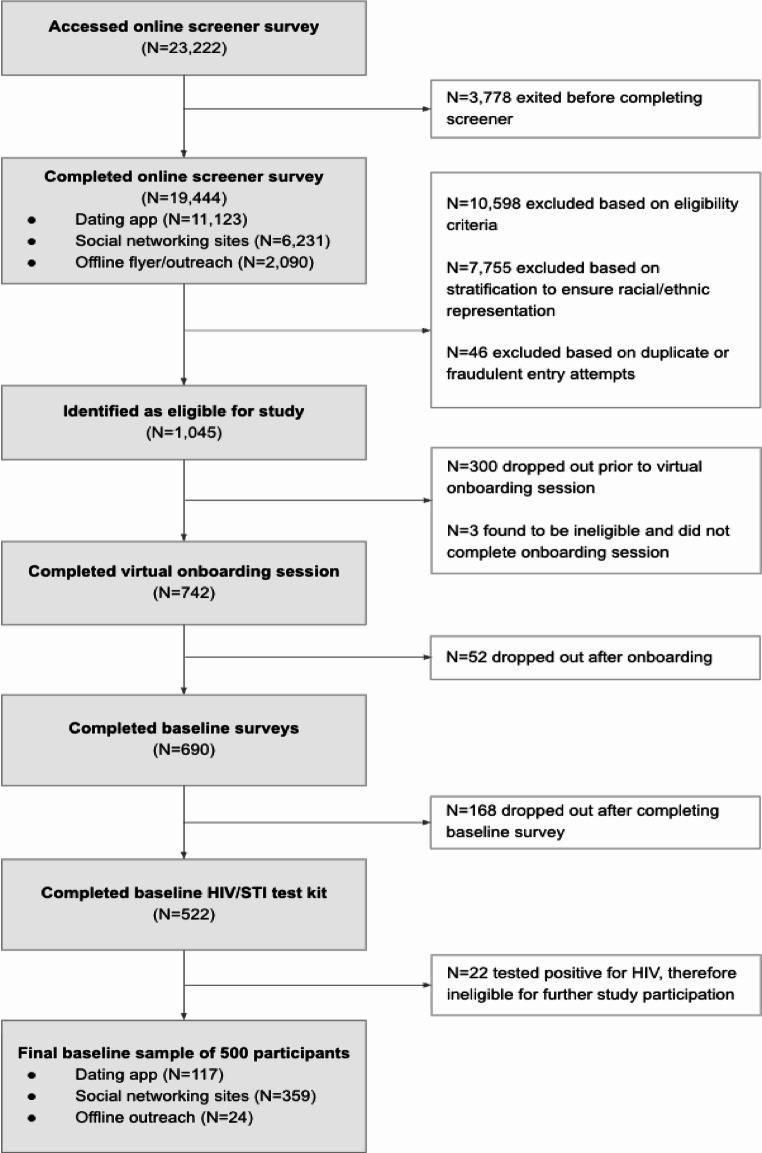
Flow chart of EROS participant recruitment

Recruitment took place between September 2022 and December 2023 via online platforms to engage men across the U.S., including social networks like Facebook and Instagram, dating and hookup apps such as Scruff, Jack’d, and Grindr, as well as through physical outreach with flyers at LGBTQ+ spaces, bars, gyms, and Pride events in the Los Angeles and San Diego regions of California (see Fig. 1 for participant recruitment flow chart). Interested individuals were directed to an online survey where they received detailed study information, consented to eligibility screening, and answered qualifying screening questions.

Eligible participants were cisgender men aged 18–45, who reported being in a relationship with another cisgender man for at least three months, being HIV-negative or having unknown status, living in a CDC-designated Ending the HIV Epidemic priority jurisdiction, and who were willing to complete mailed home-based HIV/STI test kits. These criteria were selected with the goal of recruiting men with heightened risk of exposure to both IPV and HIV. To prevent violence risk, only one partner per couple was allowed to participate, verified through partner identification details.

 Eligible participants completed a series of steps. During a live virtual onboarding session, individual participants met with a study associate who verified participant identity, explained study activities, answered participant questions, and elicited informed consent for participation. Consented participants were then provided a unique link to complete a comprehensive baseline survey and were sent a home-based biomarker kit that collected samples for HIV and STI screening and, when PrEP use was reported, to assess for PrEP adherence levels. Participants were required to complete the baseline assessment and return the test kit with viable specimen, to confirm HIV-negative status, before being fully enrolled in the study. Participants received a $50 e-gift certificate upon completion of these baseline tasks. Figure  1 shows that of the 19,444 persons who completed the screener, 1,045 (5.37%) met the initial eligibility criteria and of those, 500 (47.85%) completed all steps and were fully enrolled into the EROS cohort and formed the analytic sample for these analyses.

### Measures

#### Socio-demographics

Participants reported age, race/ethnicity, education level, employment status, residential location, and relationship status.

#### PrEP Use

Participants self-reported their current and past use of PrEP. Participants who reported current use of daily oral PrEP also provided dried blood spots via mail-in test kit to verify their use of PrEP. Daily oral PrEP use and medication adherence levels were measured using intracellular tenofovir-diphosphate levels (TFV-DP). For the purposes of the current analysis, participants with verified protective TFV-DP levels of daily oral PrEP, or who self-reported current use of on-demand (e.g. 2-1-1) oral or current use of long-acting injectable PrEP were combined and categorized as “PrEP users”; all others were considered “non-PrEP users”. Therefore, if someone self-reported daily oral PrEP, but did not have a protective level of TFV-DP, they were classified as a non-PrEP user.

#### Intimate Partner Violence Perpetration

IPV perpetration was assessed using 62 items adapted from a validated IPV measure [38], questioning whether specific behaviors were enacted towards an intimate partner during the past six months. Participants were asked to exclude behaviors associated with consensual sexual activities or accidents. Eight forms of IPV perpetration were assessed: controlling (e.g., preventing your partner from seeing their family or friends), emotional (e.g. telling your partner they are worthless), financial (e.g., preventing partner from accessing money), SMM identity-related (e.g., threatening to out a partner to family or coworkers; telling partner to act straight), physical (e.g., hitting, pushing or shoving your partner), sexual (e.g., forcing or pressuring your partner to do something sexually that they didn’t want to do), sexual health (e.g., interfering with use of PrEP or HIV testing), and stalking (e.g., following partner around and watching them when they did not want you to). A single item endorsed for a given form of IPV perpetration was scored as evidence for that form of IPV perpetration. Follow up items asked if endorsed items happened once or more than once. This measure showed 93.6% completeness.

#### Internalized Homonegativity

Internalized homonegativity was measured using a seven-item scale designed to capture the internal conflict between same-sex attraction or desires and the perceived need to conform to heterosexual norms [39]. Participants rated their agreement with items on a scale from 1 (strongly agree) to 5 (strongly disagree). Examples of statements included “I feel comfortable being seen in public with an obviously gay person” and “I feel comfortable discussing homosexuality in a public situation.” Scores for each item were summed to create a total internalized homonegativity score, with higher scores indicating greater levels of internalized homonegativity. This measure showed 97.2% completeness.

#### Anticipated Identity Stigma

To evaluate anticipatory stigma, six items assessing expectations of rejection and discrimination based on one’s identities were assessed [40]. Participants responded to these items on a four-point Likert scale ranging from 1 (Strongly Disagree) to 4 (Strongly Agree). Example items included: “Most employers will not hire a person like me,” “Most people think less of a person like me,” and “Most people look down on people like me.” We computed mean item scores for each participant, which ranged from 1 to 4, with higher scores reflecting greater anticipated stigma. This measure showed 95% completeness.

#### Experienced Sexual Orientation Discrimination

Participants’ experiences of discrimination based on sexual orientation over the past six months were assessed using an adapted version of the 10-item Multiple Discrimination Scale [41]. This adaptation was tailored to capture the specific experiences of SMM, focusing on various forms of discriminatory interactions. Example items include being “treated with hostility or coldness by strangers,” “ignored, excluded, or avoided by people close to me,” and having “someone act as if I could not be trusted.” Responses were recorded on a dichotomous scale (1 = Yes; 0 = No) and summed to produce a total score ranging from 0 to 10, with higher scores indicating more frequent discrimination experiences. The scale was 100% complete.

#### PrEP Stigma

We assessed PrEP-related stigma using the HIV PrEP Stigma Scale [42], which was developed to address multifaceted stigma barriers identified in the literature. The scale captures three key domains: internalized stigma (shame about PrEP use), anticipated stigma (expected character judgments from others), and experienced stigma (experienced character judgments from others). The HIV PrEP Stigma Scale is designed for broad applicability, making it suitable for diverse contexts such as implementation science and clinical trials, by focusing on essential stigma attributes without extensive burden on respondents [42]. This measure was 81% complete.

### Data Analysis

Descriptive statistics were used to characterize the sample’s socio-demographics and experiences related to different forms of IPV perpetration, minority stress constructs of interest, and PrEP use. Bivariate analyses (using two-tailed independent t-tests and Pearson correlations) were conducted to examine relationships between various forms of IPV perpetration, PrEP use, and four minority stress constructs that may act as mediators (internalized homonegativity, anticipatory identity stigma, sexual orientation discrimination, and PrEP-related stigma). Missing data were handled using specific criteria for different measures. For scales assessing internalized homonegativity, anticipatory stigma, and PrEP-related stigma, mean values were imputed as the average of non-missing items if fewer than half of the items on the measure were missing. If half or more of the items were missing, the entire scale was treated as missing. For scales with an odd number of items, the half benchmark was calculated as $\:\frac{t-1}{2}$, where *t* was the total number of items in a scale. In the case of sexual orientation discrimination and IPV perpetration measures, individual missing items were imputed as 0; however, if all items were missing, the overall scale was set to missing. For PrEP use, no imputation was performed for missing values. This approach aimed to balance maximizing available data while maintaining the integrity of the measures across different constructs.

To examine the potential mediating roles of internalized homonegativity, anticipatory stigma, sexual orientation discrimination, and PrEP-related stigma in the relationship between IPV perpetration and PrEP use, we employed a series of logistic regression models. First, we tested to ensure the assumptions were met for logistic regression. To test for multicollinearity, correlations were calculated for each pair of independent variables, and there were no signs of violation of this assumption. Further, the assumption of linearity of the log-odds outcome and the continuous independent variables were assessed by independently graphing the outcome (PrEP use) against each continuous explanatory variable (age, internalized homonegativity, experienced sexual orientation discrimination, PrEP stigma, and anticipated identity stigma) plus its residuals. The results demonstrated linear, monotonic lines suggesting the continuous explanatory variables were linear with the log-odds of PrEP use. We also assessed for outlier effects and did not find any of concern. We then ran a series of logistic regression models. We first evaluated the direct association between each type of IPV perpetration and PrEP use. Subsequently, we tested four separate mediation models, each incorporating one of the four hypothesized mediators. To qualify for mediation analysis, a significant (*p* <.05) association was required between (1) IPV perpetration type and the mediator; and (2) the mediator and PrEP use [43, 44]. 

For each mediation model, we followed a two-step process. First, we estimated a logistic regression model predicting PrEP use from IPV perpetration type alone. A statistically significant effect suggests that the effect of a mediator can be further investigated. Second, we estimated the same model while including the hypothesized mediator. We then compared the magnitude of the association between IPV perpetration type and PrEP use across the two models to assess whether the mediator significantly altered the relationship. Given the potential for intercorrelations among mediators, a final model was also constructed incorporating the mediators simultaneously. All mediation models included age, education, and race/ethnicity as covariates to control for potential confounding factors.

In the models, the **direct effect** represents the association between identity-related IPV perpetration and PrEP use, independent of any influence from the mediator. A significant direct effect suggests that the relationship between IPV perpetration and PrEP use persists even when accounting for the mediator, indicating that the mediator does not fully explain the association. In contrast, the **indirect effect** reflects the portion of this relationship that is explained by the mediator. A significant indirect effect indicates that the mediator accounts for a meaningful part of the link between IPV perpetration and PrEP use. For full mediation to be established, the indirect effect must be significant, while the direct effect should not be. This pattern would suggest that the relationship between identity-related IPV perpetration and PrEP use operates entirely through the mediator. We conducted significance testing of direct and indirect effects using a bootstrap approach with 1,000 samples. SPSS Statistics version 29 and R Studio version 2024.04.2 + 764 were used for data analysis.

## Results

### Participant Characteristics

Table 1 presents the demographic characteristics of the 500 SMM enrolled in the study, including information related to IPV perpetration type, PrEP use, and the minority stress factors examined as mediators in the analysis. The sample was geographically diverse, residing across all Ending the HIV Epidemic identified high-priority jurisdictions [45]. The mean age was 32.7 years (SD = 6.2), with 72.6% reporting completion of at least four years of college. The majority of participants were currently employed (81.0%) and in a relationship (88.8%). While all participants reported being in a relationship at eligibility screening, several were no longer in a relationship by the time they completed the baseline survey. Racial and ethnic diversity was also evident, with 30.0% of participants identifying as non-Hispanic White, 34.2% as Hispanic or Latino, 16.8% as non-Hispanic Black, and 14.6% as non-Hispanic Asian or Pacific Islander.

**Table 1:** Sample characteristics by use of PrEP (*N* = 500)

	Total (*N* = 500)	PrEP User (*n* = 212)	Non-PrEP User (*n* = 288)	
Sociodemographic characteristics	M (SD)	M (SD)	M (SD)	p-value
Age	32.7 (6.2)	33.4 (6.2)	32.2 (6.3)	0.04
	n (%)	n (%)	n (%)	p-value
Race/ethnicity				0.04
White	150 (30.0%)	77 (36.3%)	73 (25.3%)	
Black/African American	84 (16.8%)	37 (17.5%)	47 (16.3%)	
Hispanic/Latino	171 (34.2%)	67 (31.6%)	104 (36.1%)	
Asian/Pacific Islander	73 (14.6%)	22 (10.4%)	51 (17.7%)	
Multiracial/Other	22 (4.4%)	9 (4.2%)	13 (4.5%)	
At least 4 years of college	363 (72.6%)	163 (76.9%)	200 (69.4%)	0.06
Employed	405 (81.0%)	175 (82.5%)	230 (79.9%)	0.45
Region of residence				0.32
Northeast	118 (23.6%)	53 (25.0%)	65 (22.6%)	
South	165 (33.0%)	76 (35.8%)	89 (30.9%)	
West	149 (29.8%)	54 (25.5%)	95 (33.0%)	
Midwest	68 (13.6%)	29 (13.7%)	39 (13.5%)	
Reside in urban location	121 (24.2%)	54 (25.7%)	67 (23.3%)	0.60
Currently in a relationship	444 (88.8%)	184 (87.7%)	260 (91.0%)	0.25
IPV perpetration				
Any perpetration of IPV	125 (25%)	53 (25.0%)	72 (25.1%)	0.98
	M (SD)	M (SD)	M (SD)	p-value
Any IPV perpetration occurring more than once^a^	1.5 (2.54)	1.68 (3.32)	1.36 (1.77)	0.53
Any IPV perpetration type occurring more than once^a^	0.96 (1.03)	0.91 (0.97)	1.00 (1.09)	0.61
Controlling perpetration^a^	0.63 (0.87)	0.81 (1.06)	0.50 (0.67)	0.06
Emotional perpetration^a^	0.81 (1.13)	1.06 (1.38)	0.63 (0.86)	0.05
Financial perpetration^a^	0.06 (0.25)	0.08 (0.27)	0.06 (0.23)	0.66
Identity-related perpetration^a^	0.13 (0.42)	0.02 (0.14)	0.21 (0.53)	< 0.01
Physical perpetration^a^	0.90 (1.51)	0.77 (1.59)	0.99 (1.46)	0.45
Sexual perpetration^a^	0.14 (0.40)	0.11 (0.38)	0.17 (0.41)	0.45
Sexual health perpetration^a^	0.10 (0.31)	0.09 (0.30)	0.11 (0.32)	0.76
Stalking perpetration^a^	0.29 (0.72)	0.30 (0.89)	0.28 (0.57)	0.89
Repeated identity-related perpetration^a^	0.07 (0.26)	0.02 (0.14)	0.11 (0.32)	< 0.05
Potential mediators				
Internalized Homonegativity	12.8 (4.4)	12.02 (4.3)	13.4 (4.5)	< 0.001
Anticipated Identity Stigma	1.8 (0.7)	1.7 (0.6)	1.8 (0.7)	0.13
Sexual Orientation Discrimination	0.6 (1.1)	0.6 (1.1)	0.5 (1.2)	0.60
PrEP Stigma	2.02 (0.6)	1.8 (0.5)	2.2 (0.6)	< 0.001

^a^*M* Mean, *SD* Standard deviation. Means and standard deviations were calculated among participants who reported any perpetration of IPV, not among the whole sample

### PrEP Use

Of the total sample, 42.4% (*n* = 212) reported using PrEP. Characteristics such as education, employment, relationship status, and residence location were similar between PrEP users and non-PrEP users. PrEP users were slightly older (M = 33.4, SD = 6.2) than non-PrEP users (M = 32.2, SD = 6.3, *p* <.05). More PrEP users identified as White than non-PrEP users (36.3% vs. 25.3%), whereas Asian and Hispanic/Latino participants were more likely to be non-PrEP users than PrEP users (17.7% vs. 10.4% and 36.1% vs. 31.6%, respectively, *p* <.05). Internalized homonegativity scores were significantly higher among non-PrEP users (M = 13.4, SD = 4.5) than among PrEP users (M = 12.02, SD = 4.3; *p* <.001). PrEP stigma scores were also significantly higher among non-PrEP users (M = 2.2, SD = 0.6) than among PrEP users (M = 1.8, SD = 0.05; *p* <.001).

### IPV Perpetration

One hundred twenty-five participants (25%) reported at least one behavior of IPV perpetration with a main partner in the prior 6 months. Table 1 presents each IPV perpetration type for the overall sample and by use of PrEP. Among those reporting any IPV perpetration, the mean number of IPV perpetration behaviors that occurred more than once in the prior six months was 1.50 (SD = 2.54; median = 1.0; IQR = 2). The mean number of IPV perpetration forms in which at least one behavior type occurred more than once was 0.96 (SD = 1.03; median = 1.0; IQR = 1). The highest mean numbers of IPV perpetration type among those reporting any IPV perpetration were controlling (M = 0.63, SD = 0.87), emotional (M = 0.81, SD = 1.13), and physical IPV (M = 0.90, SD = 1.51). The mean number of identity-related IPV perpetration items among those who reported any form of perpetration was 0.13 (SD = 0.42).

### IPV Perpetration and PrEP Use

Of all IPV perpetration forms, identity-related IPV was the only form found to be significantly associated with current PrEP use. Non-PrEP users reported significantly more perpetration of identity-related IPV (M = 0.21, SD = 0.53) than PrEP users (M = 0.02, SD = 0.14, *p* <.01). Moreover, non-PrEP users reported significantly more instances of repeated perpetration of identity-related IPV (M = 0.11, SD = 0.32) than PrEP users (M = 0.02, SD = 0.14, *p* <.05). PrEP users and non-PrEP users did not differ significantly on other forms of IPV perpetration.

### Minority Stress Correlates with IPV-Identity Perpetration and PrEP Use

The correlations between the potential minority stress mediators (anticipated identity stigma, experienced sexual orientation discrimination, internalized homonegativity, and PrEP stigma) with IPV perpetration and PrEP use are presented in Table 2. Anticipated identity stigma and experienced sexual orientation discrimination were not significantly correlated with PrEP use. However, experienced sexual orientation discrimination was significantly correlated with perpetration of identity-related IPV (*r* =.15, *p* <.01). PrEP use was significantly correlated with internalized homonegativity (*r*=-.16, *p* <.001) and PrEP stigma (*r* = −.33, *p* <.001). Perpetration of identity-related IPV was significantly correlated with each of the potential minority stress mediators, except for anticipated identity stigma; therefore, anticipated identity stigma was not included in the mediation analysis. Table 3 presents correlations among each of the potential mediators. PrEP stigma and internalized homonegativity were correlated with each other (*r* =.43, *p* <.001), suggesting the need for a single mediation model that includes both mediators.

**Table 2:** Bivariate correlates of PrEP use and IPV perpetration with potential mediators

	Current PrEP use	Any IPV perpetration	IPV-Identity perpetration	Repeated IPV-Identity perpetration
Anticipated identity stigma	− 0.07	0.04	0.03	0.03
Experienced sexual orientation discrimination	0.02	0.19***	0.15**	0.16***
Internalized homonegativity	− 0.16***	0.06	0.19***	0.17***
PrEP stigma	− 0.33***	0.15**	0.14***	0.15***

*p <.05; **p <.01; ***p <.001

**Table 3:** Bivariate correlates among potential mediators

	Anticipated identity stigma	Experienced sexual orientation discrimination	Internalized homo-negativity	PrEP stigma
Anticipated identity stigma	1	0.30***	0.27***	0.25***
Experienced sexual orientation discrimination		1	0.06	0.04
Internalized homonegativity			1	0.43***
PrEP stigma				1

*p <.05; **p <.01; ***p <.001

### Mediation Analysis

An initial logistic regression model was run to assess the association between perpetration of identity-related IPV and PrEP use without any mediators, adjusting for age, race/ethnicity, and education level as these were the covariates found to be at least marginally associated with PrEP use (see Table 4). The results show that those who perpetrated identity-related IPV had significantly lower odds of being a PrEP user (OR = 0.13, 95% CI = 0.01, 0.63). Next, to assess any potential effects of the mediators, each potential mediator (i.e., experienced sexual orientation discrimination, internalized homonegativity, and PrEP stigma) was examined in separate logistic regression models. Experiencing sexual orientation discrimination was found to not mediate the relationship between perpetration of identity-related IPV and PrEP use as evidenced by a non-significant indirect effect (OR = 1.03, 95% CI = 0.95, 1.13)] and a significant direct effect (OR = 0.30, 95% CI = 0.10, 0.86). Internalized homonegativity was found to fully mediate the relationship between perpetration of identity-related IPV and PrEP use as evidenced by a significant indirect effect (OR = 0.88, 95% CI = 0.78, 0.98) and a non-significant direct effect (OR = 0.35, 95% CI = 0.12, 1.01). PrEP stigma was shown to also fully mediate the relationship between perpetration of identity-related IPV and PrEP use as evidenced by a significant indirect effect (OR = 0.77, 95% CI = 0.60, 0.98) and non-significant direct effect (OR = 0.40, 95% CI = 0.15, 1.10). Since internalized homonegativity and PrEP stigma were found to be correlated with each other, these two mediators were then added into one model to assess the effect of the two mediators together. Together, internalized homonegativity and PrEP stigma only partially mediated the relationship between perpetration of identity-related IPV and PrEP use, where both the indirect effect (OR = 0.78, 95% CI = 0.60, 1.00) and the direct effect (OR = 0.39, 95% CI = 0.14, 1.08) of IPV-identity perpetration on PrEP use were no longer significant. Moreover, the effect of internalized homonegativity on PrEP use disappeared (OR = 1.01, 95% CI = 0.98, 1.03), while the effect of PrEP stigma on PrEP use remained significant (OR = 0.47, 95% CI = 0.39, 0.57). Each model adjusted for age, education level and race/ethnicity. None of these covariates were found to be significant in any of the models.

**Table 4:** Logistic regression mediation analysis predicting PrEP use

	No mediator Odds Ratio (95% CI)	Mediator: experienced sexual orientation discrimination Odds Ratio (95% CI)	Mediator: Internalized Homonegativity Odds Ratio (95% CI)	Mediator: PrEP Stigma Odds Ratio (95% CI)	Mediators: Internalized Homonegativity PrEP Stigma Odds Ratio (95% CI)
Identity-related IPV Perpetration	0.13 (0.01–0.63)	0.13 (0.01–0.63)	0.13 (0.01–0.63)	0.13 (0.01–0.63)	0.13 (0.01–0.63)
Direct effect	–	0.30 (0.10–0.86)	0.35 (0.12–1.01)	0.40 (0.15–1.10)	0.39 (0.14–1.08)
Potential Mediators					
Experienced sexual orientation discrimination	–	1.04 (0.94–1.15)	–	–	–
Internalized homonegativity	–	–	0.97 (0.94–0.99)	–	1.01 (0.98–1.03)
PrEP Stigma	–	–	–	0.48 (0.40–0.57)	0.47 (0.39–0.57)
Indirect effect	–	1.03 (0.95–1.13)	0.88 (0.78–0.98)	0.77 (0.60–0.98)	0.78 (0.60–1.00)
Covariates					
Age	1.03 (1.00–1.06)	1.02 (1.00–1.03)	1.02 (1.00–1.03)	1.02 (1.00–1.03)	1.01 (1.00–1.03)
Hispanic or Multiracial Hispanic	0.96 (0.38–2.46)	0.98 (0.55–1.75)	0.99 (0.55–1.76)	0.99 (0.55–1.76)	0.99 (0.56–1.78)
Non-Hispanic White	1.41 (0.57–3.65)	1.24 (0.69–2.23)	1.24 (0.69–2.23)	1.26 (0.70–2.25)	1.26 (0.70–2.25)
Non-Hispanic Black or Multiracial Black	1.11 (0.42–3.00)	1.07 (0.58–1.98)	1.07 (0.58–1.98)	1.08 (0.58–2.00)	1.08 (0.59–2.01)
Asian/Pacific Islander (API) or Multiracial API	0.55 (0.20–1.53)	0.70 (0.37–1.30)	0.70 (0.37–1.30)	0.70 (0.37–1.30)	0.70 (0.37–1.30)
Education (at least 4 years of college vs. less)	1.45 (0.95–2.22)	1.26 (0.97–1.64)	1.26 (0.97–1.64)	1.26 (0.97–1.64)	1.26 (0.97–1.65)

*CI* Confidence interval

## Discussion

Our findings illuminate the complex interplay between internalized homonegativity, PrEP stigma, IPV perpetration, and PrEP use among cisgender SMM. We identified a significant association between perpetration of identity-related IPV and PrEP use. This relationship was independently mediated by internalized homonegativity and PrEP stigma, suggesting that the association between perpetrating identity-based IPV and PrEP use can be explained by either internalized homonegativity or PrEP stigma. These findings suggest that SMM who report perpetrating identity-related IPV may be less likely to use PrEP, potentially due in part to higher levels of internalized homonegativity and PrEP-related stigma. Of the two, PrEP stigma was the stronger mediator, showing a significant indirect effect and a non-significant direct effect. This may suggest that PrEP stigma is more proximal to PrEP uptake, as the stigma specifically targets the use of PrEP itself.

These results are consistent with Minority Stress Theory [29, 30], which posits that chronic stressors (e.g., prejudice, discrimination, violence, internalized stigma, anticipation of rejection) faced by marginalized groups, such as sexual minority individuals, contribute to poorer health outcomes and suboptimal health service utilizations [25]. Prior work has shown that internalized homonegativity [12], in particular, can serve as an impediment to PrEP engagement among young SMM [31]. Our findings extend prior work that has shown minority stressors to be associated with IPV generally [32], and provides further support for the psychological mediation framework [13, 33, 34], by demonstrating that internalized homonegativity and PrEP stigma can mediate the relationship between IPV perpetration and PrEP use. Indeed, our findings suggest that both internalized homonegativity and PrEP-related stigma appear to play a role in the link between perpetration of identity-related IPV and non-PrEP use. This reinforces the important need for public health policies and interventions that address both the societal stigma and the internalized stressors surrounding sexual minority identity and PrEP use.

Our findings suggest that SMM who are less comfortable with their own sexuality are also more likely to hold negative attitudes about the use of PrEP and be less likely to take it themselves, as PrEP use is often associated with same-sex behavior. Moreover, these same individuals may also act out against their partner’s sexual minority identity and serve as barriers to their partners’ use of PrEP. This finding has important implications for understanding HIV transmission risk within this population, as individuals perpetrating identity-related IPV may be at a particularly high risk for HIV acquisition due to their lower likelihood of using PrEP, and their partners may also be at elevated risk.

At a structural level, social media campaigns and provider training programs can reduce PrEP stigma by normalizing PrEP use within both heterosexual and sexual minority communities, and by promoting PrEP as a responsible, prosocial strategy for reducing HIV transmission [25, 46, 47]. The persistence of PrEP stigma more than a decade after its introduction suggests an ongoing need to directly address stereotypes associated with PrEP use.

Additionally, policies that expand access to affirming, comprehensive sexual health and IPV services for SMM of all sexual identities are essential [7]. Addressing structural determinants such as provider bias and discrimination in healthcare settings are also key to creating an environment that does not reinforce PrEP stigma [47–49]. At the individual level, culturally responsive IPV and sexual health assessments and behavioral interventions are needed to help SMM process and challenge internalized homonegativity, while also promoting PrEP-related health literacy and self-efficacy [7, 16]. Motivational interviewing [50, 51], cognitive-behavioral strategies [25, 52], and peer-led PrEP navigation [53, 54] models have shown promise in reducing PrEP stigma, increasing demand, and enhancing uptake. These intervention strategies could be integrated into existing sexual health education, services and IPV prevention programs for SMM.

Our findings highlight the importance of focusing on the perpetration of identity-related IPV as a key target for HIV prevention and intervention efforts. Behaviors like threatening to out a partner to family or coworkers, demanding that a partner act ‘straight,’ and criticizing a partner based on race or ethnicity are not only directly harmful to the receiving partner and the relationship, but may also be an indication of significant barriers to PrEP use. Our findings extend prior literature suggesting that perpetration of identity-related IPV and non-use of PrEP may both reflect internalized homonegativity and PrEP stigma, which likely stems from the perpetrator’s own discomfort with their sexual orientation.

Future research is needed to further explore the mechanisms by which internalized homonegativity and PrEP stigma mediate the relationship between identity-related violence in relationships and PrEP use, and to develop and evaluate interventions specifically designed to address internalized homonegativity, PrEP stigma, reduce perpetration of identity-related IPV, and promote PrEP use among SMM. Although internalized homonegativity and PrEP stigma were identified as mediators in this relationship, other unmeasured factors likely contribute to the link between perpetration of identity-related IPV and PrEP use. Potential factors could include experiences of broader social and structural marginalization (e.g., racism, economic marginalization, disparate incarceration) [55, 56], mistrust of healthcare providers or settings [57], unmet mental health needs related to depression, PTSD, or anxiety [58], and access to health care resources [59, 60]. Additionally, future studies should explore the bidirectionality of IPV within relationships, considering how experiences of homophobia and homonegative attitudes may create relationship strain, potentially leading partners to direct frustrations towards each other. This cyclical nature of violence and its impact on PrEP use behaviors warrants further investigation. Investigating the role of positive social support networks and connectedness to the larger sexual and gender diverse community in mitigating the effects of IPV and promoting PrEP use among SMM is also crucial.

Public health and IPV prevention interventions should consider integrating screening for IPV perpetration and victimization into routine sexual health care for SMM, particularly those who are at higher risk. This also includes the need for a brief, culturally appropriate IPV screening tool in clinical settings to ensure accurate and timely identification of IPV among SMM. Offering resources and support services for SMM experiencing IPV, including access to confidential counseling, safety planning, and legal advocacy, is also essential. Additionally, it is crucial to develop and implement trauma-informed, healing-centered services that address the perpetration of IPV among SMM, recognizing that for many, perpetration may stem from their own experiences of homonegativity, victimization, and trauma [14, 61]. Meta analyses suggest that perpetration interventions that address substance use and trauma are more effective [62]. Interventions that incorporate motivational interviewing techniques also show evidence of effectiveness for engagement and outcomes [63]. While evidence is mixed, bystander interventions [64, 65], as recommended by the CDC [66], could be adapted for SMM communities to identify the signs and further prevent IPV. Engaging peers and educating community members to be able to identify the signs of IPV and provide support to help interrupt the perpetration of IPV may help to promote healthy relationships among SMM. Promoting comprehensive sexual health education that addresses the unique challenges faced by SMM, including the intersection of IPV, sexual orientation, and HIV prevention, is crucial. Advocating for policies that protect SMM from discrimination and promote access to affordable and comprehensive healthcare, including PrEP, is vital.

The present study was not without limitations. First, the cross-sectional nature of the data precluded any causal inferences about the relationships between variables. Also, logistic regression may overestimate the prevalence ratio when using cross-sectional data. Although the sample size was large and diverse overall, the subgroup of participants reporting identity-based IPV perpetration was relatively small. This may have resulted in limited statistical power to detect significant associations between perpetration of identity-related IPV and the other variables examined in these analyses. Moreover, the study did not assess whether IPV perpetration occurred in response to the need for self-defense, which is a factor that could influence the interpretation of these behaviors. Because participants were asked to report IPV perpetration with any main partner during the past 6-months, it is possible that IPV may have occurred with a main partner other than the main partner they reported at baseline. Additionally, while online recruitment facilitated the inclusion of participants from across the United States, it may have inadvertently biased the sample toward individuals with greater access to technological resources and privacy in their home. Conversely, our flyer-based recruitment efforts, which could potentially reach men of lower socioeconomic status, were geographically restricted to the Los Angeles and San Diego areas. These recruitment strategies may limit the generalizability of the current findings to broader populations. Finally, while mean imputation is a commonly used and valid approach for handling missing data, we acknowledge that this method may artificially inflate correlations between variables by reducing variability. Future analyses using more robust imputation methods (e.g., multiple imputation) may help to confirm the stability of these findings.

To conclude, understanding the complex interplay between IPV perpetration, PrEP use, and internalized homonegativity is critical for effectively addressing HIV risk among SMM. Focusing on perpetration of identity-based IPV, internalized homonegativity, and promoting access to PrEP are key components of a multi-pronged approach to HIV prevention and intervention within this population.
